# Cardiac Amyloidosis in the Real World: Clinical Presentations, Disease Overlap, and Therapeutic Imperatives

**DOI:** 10.31083/RCM42514

**Published:** 2025-10-20

**Authors:** Karim Ali, Ahmed E. Ali, Ahmad Alayyat, Mohamed K. Awad, Hussain Majeed, Mohamed S. Amer, Ahmed Sami Abuzaid

**Affiliations:** ^1^Department of Internal Medicine, Hennepin County Medical Center, Minneapolis, MN 55415, USA; ^2^Department of Internal Medicine, Crestwood Medical Center, Huntsville, AL 35801, USA; ^3^Department of Internal Medicine, Hamilton Medical Center, Dalton, GA 30720, USA; ^4^Department of Critical Care, Faculty of Medicine, Ain Shams University, 11646 Cairo, Egypt; ^5^Department of Internal Medicine, Northeast Georgia Medical Center, Gainesville, GA 30501, USA; ^6^Department of Cardiology, El Mansoura International Hospital, 35516 Mansoura, Egypt; ^7^Alaska Heart and Vascular Institute, University of Alaska, Anchorage, AK 99571, USA

**Keywords:** cardiac amyloidosis, transthyretin amyloidosis, AL amyloidosis, heart failure with preserved ejection fraction, aortic stenosis, cardiac magnetic resonance imaging, bone scintigraphy, artificial intelligence in cardiology, amyloid biomarkers, endomyocardial biopsy002E

## Abstract

Cardiac amyloidosis (CA) has emerged from the margins of cardiology to the forefront of research and practice on heart failure. Once regarded as rare and elusive, CA is now recognized as a significant cause of heart failure with preserved ejection fraction (HFpEF), arrhythmias, and valvular disease, especially in older patients. CA is characterized by extracellular deposition of misfolded protein fibrils, which infiltrate the myocardium and disrupt the structural and electrical integrity. Although CA can stem from multiple amyloid types, transthyretin amyloidosis (ATTR) and light-chain (AL) amyloidosis are the predominant subtypes with cardiac involvement, each carrying distinct implications for prognosis and therapy. This review explores CA as a clinical reality often obscured by more common cardiovascular syndromes. Moreover, this review focuses on the varied presentations of CA in real-world practice, how the condition overlaps with HFpEF, the subtle clues for CA amid common valvular disorders, and the complex rhythm manifestations of the condition. Particular attention is given to thromboembolic risk, microvascular dysfunction, and the evolving paradigm of preclinical or asymptomatic amyloidosis management. Furthermore, this review addresses contemporary challenges such as financial toxicity and the cost-effectiveness of screening, emphasizing the benefits of early detection and therapy. The paper also discusses risk stratification and staging, drawing from validated models to guide both prognosis and treatment decisions, and the role of histopathological characterization. Thus, this review underscores the importance of timely recognition and tailored intervention in transforming CA from a terminal diagnosis into a manageable chronic condition.

## 1. Risk Features, Staging, and Screening Cardiac Amyloidosis (CA)

Numerous clinical, laboratory, and imaging findings, ranging from extracardiac “red flags” like carpal tunnel syndrome to specific electrocardiographic and echocardiographic changes, help identify CA early [1, 2], as summarized in Table 1. Notably, longitudinal strain, transmural late gadolinium enhancement (LGE), myocardial extracellular volume (ECV), and hepatic ECV in systemic light-chain (AL) amyloidosis are independently associated with increased mortality [3, 4]. After diagnosis, staging using established systems such as Mayo (2004/2012) for AL amyloidosis and the National Amyloidosis Centre (NAC) criteria for transthyretin amyloidosis (ATTR) guide prognosis and therapy [5, 6]. While newer systems (e.g., Europeanmodified Mayo) have improved stratification in research settings, they remain secondary in routine clinical care [7].

**Table 1.** **Alarming features in cardiac amyloidosis (CA)**.

Domain	Alarming features
Genetics	- AL amyloidosis: IGLV1-44 germline gene
	- ATTR amyloidosis: p.V142I, p.T80A, p.V50M
Clinical Picture	- Male sex, age >60
	- Family history of neuropathy or unexplained heart failure
	- HFpEF with normal blood pressure
	- Atrial fibrillation without other causes
	- Intolerance to GDMT
	- Orthostatic hypotension or syncope
	- Diagnosed HCM after age 60
	- Low-flow, low-gradient AS
	- Unexplained AV block, bradycardia, or pacemaker implantation
	- Bilateral carpal tunnel syndrome
	- Lumbar spinal stenosis
	- Peripheral/autonomic neuropathy
	- Macroglossia or altered taste
	- Recurrent pleural effusions
	- History of plasma cell disorder
Laboratory	- Elevated troponin T or I
	- Elevated NT-proBNP
	- Nephrotic-range proteinuria
Electrocardiography (ECG)	- Low QRS voltage
	- Pseudoinfarction pattern (e.g., Q waves without coronary artery disease)
Echocardiography	- Increased LV wall thickness
	- Apical sparing pattern of longitudinal strain
	- Right ventricular hypertrophy
	- Biatrial enlargement
	- Restrictive filling pattern
	- Pericardial effusion
	- Atrial septal and valvular thickening
	- Granular sparkling myocardium
Other Imaging	- Transmural LGE on CMR
	- Elevated myocardial or hepatic extracellular volume (ECV)
	- Cardiac uptake on bone scintigraphy (DPD/PYP grade 2–3)
	- Amyloid PET tracer uptake (e.g., 18F-florbetapir)

AL, light-chain; ATTR, transthyretin amyloidosis; HFpEF, heart failure with preserved ejection fraction; GDMT, guideline-directed medical therapy; HCM, hypertrophic cardiomyopathy; AS, aortic stenosis; AV, atrioventricular; NT-proBNP, N-terminal pro–B-type natriuretic peptide; ECG, electrocardiography; LGE, late gadolinium enhancement; CMR, cardiac magnetic resonance; DPD/PYP, bone scintigraphy tracers; PET, positron emission tomography; LV, left ventricular.

### 1.1 Early Biopsy & Non-biopsy Diagnosis

Endomyocardial biopsy (EMB) remains the definitive diagnostic standard, especially in cases of suspected AL amyloidosis, monoclonal proteinemia, equivocal imaging, or coexisting conditions [8, 9]. However, in ATTR amyloidosis, particularly wild-type ATTR (wtATTR), non-biopsy diagnosis using bone-avid tracer scintigraphy (99mTc-PYP, DPD, or HMDP) combined with negative serum and urine immunofixation and serum free light chain (sFLC) assay has high specificity and is now endorsed in major guidelines [10]. Importantly, EMB is still advised if monoclonal gammopathy is present, despite highgrade tracer uptake, to avoid misclassification [11].

### 1.2 Targeted Populations

Several clinical scenarios are recognized as high-yield targets for CA screening, including older adults with bilateral carpal tunnel syndrome, a condition that often precedes clinical CA by approximately 5 to 15 years [12]. Similarly, older patients diagnosed with heart failure with preserved ejection fraction (HFpEF) warrant evaluation, as up to 13% may exhibit significant tracer uptake indicative of CA [13]. Additionally, patients with aortic stenosis (AS), especially those undergoing evaluation for transcatheter aortic valve replacement (TAVR), show a substantial prevalence (approximately 9–16%) of coexisting CA [14]. We propose that patients with severe AS who are candidates for valve replacement should undergo evaluation using the RAISE score, followed by bone scintigraphy if found to be high-risk (Table 2) [15]. Screening is also recommended for individuals presenting with plasma cell disorders, neuropathy, indications for pacemaker implantation, lumbar spinal stenosis, or recurrent tendon ruptures due to their increased risk of underlying cardiac amyloid deposition [16].

**Table 2.** **RAISE score for screening cardiac amyloidosis (CA) in patients referred for TAVR**.

RAISE component	Points
Left ventricular hypertrophy and/or diastolic dysfunction	1
Age >85 years	1
High-sensitivity troponin I >20 ng/L	1
History of carpal tunnel syndrome	3
Right bundle branch block	2
Low QRS voltage on ECG	1

TAVR, transcatheter aortic valve replacement.

### 1.3 Screening Modalities

Screening for CA should be tailored to the suspected subtype. For ATTR amyloidosis, particularly in older patients with HFpEF or AS, initial evaluation should include serum and urine immunofixation and difference in free light chains (dFLC) assays to rule out monoclonal protein. If these tests are negative, technetium-labeled bone scintigraphy (e.g., DPD or PYP) should be performed [16, 17, 18]. In cases of conflicting or inconclusive results, cardiac magnetic resonance imaging (CMR) or EMB is recommended [9, 19]. For AL amyloidosis, any positive monoclonal protein testing warrants an immediate EMB, regardless of imaging findings. Once CA is confirmed, staging using validated systems such as the Mayo or NAC criteria helps guide prognosis and treatment intensity [4, 5, 6].

### 1.4 Staging Strategy

Staging systems can be helpful in CA to assess severity, guide therapy, and identify appropriate clinical trial candidates, as summarized in (Table 3) [2, 7]. For AL amyloidosis, the Mayo 2012 staging system, using biomarkers including troponin T, NT-proBNP, and sFLC, is widely validated [4, 5, 6]. Although the European modification of the Mayo system offers improved predictive accuracy in research, its broader clinical acceptance is limited due to inconsistent validation and outdated initial treatment paradigms [7]. Thus, updates reflecting contemporary treatments like bortezomib-based regimens are essential.

**Table 3.** **Commonly used staging systems for cardiac amyloidosis (CA)**.

System		Biomarker cutoffs	Staging criteria
AL			
	Mayo 2012	- NT-proBNP ≥1800 ng/L	- Stage I: All markers below threshold
		- Troponin T ≥25 ng/L	- Stage II: 1 marker elevated
		- dFLC ≥180 mg/L	- Stage III: 2 elevated
			- Stage IV: All 3 elevated
	European Modification of Mayo 2004	- NT-proBNP: 332–8500 ng/L	- Stage I: Both below cutoffs
		- Troponin T ≥35 ng/L	- Stage II: One above cutoff
			- Stage IIIa: NT-proBNP 332–8500 + elevated troponin T
			- Stage IIIb: NT-proBNP >8500 + elevated troponin T
ATTR			
	Mayo	- NT-proBNP ≥3000 ng/L	- Stage I: Both below cutoffs
		- Troponin T ≥50 ng/L	- Stage II: One above cutoff
			- Stage III: Both above cutoffs
	NAC	- NT-proBNP ≥3000 ng/L	- Stage I: Both below cutoffs
		- eGFR ≤45 mL/min/1.73 m^2^	- Stage II: One above cutoff
			- Stage III: Both above cutoffs
	Columbia	- NYHA Class I–IV: 1 point per class	- Total Score: 1–3 = Low Risk, 4–6 = Intermediate Risk, 7–9 = High Risk
		- Diuretic dose (furosemide equivalent):	
		• 0 mg/kg = 0 pts	
		• 0–0.5 mg/kg = 1 pt	
		• 0.5–1 mg/kg = 2 pts	
		• >1 mg/kg = 3 pts	
		- Base: NAC or Mayo stage	

dFLC, difference in free light chains; NT-proBNP, N-terminal pro–B-type natriuretic peptide; NAC, National Amyloidosis Centre; eGFR, estimated glomerular filtration rate; NYHA, New York Heart Association functional class.

In ATTR amyloidosis, the Mayo and NAC staging systems incorporate NT-proBNP, troponins, and renal function. The Mayo system was validated primarily in wtATTR, whereas the NAC system is applicable to both wild-type and variant ATTR amyloidosis [20, 21]. The recently developed Columbia system, integrating New York Heart Association (NYHA) functional class and diuretic dosage, has improved prognostic accuracy but requires further clinical validation [22]. Due to these limitations, clinicians must carefully interpret staging systems, underscoring the need for ongoing refinement to align with current clinical practices.

## 2. Valve Disease and Rhythm Disorders in CA

### 2.1 Valvular Involvement

#### 2.1.1 Aortic Stenosis

2.1.1.1 Prevalence, Presentation and Pathophysiology

Aortic stenosis and CA, particularly ATTR amyloidosis, often coexist in elderly patients and present a diagnostic and therapeutic challenge due to overlapping clinical and hemodynamic features. Some studies found that approximately 4–16% of patients undergoing TAVR for AS have concurrent ATTR amyloidosis [14, 23]; hence, it’s very important for clinicians to be aware of this dual pathology coexistence. A common presentation in these patients is low-flow, low-gradient severe AS with preserved left ventricular ejection fraction (LVEF), a restrictive pattern that reflects the stiff, infiltrated myocardium typically seen in CA. These patients often exhibit a low stroke volume index (SVi), reduced myocardial contraction fraction (MCF), low mitral annular S’ velocity, and low tricuspid annular S’ velocity distinguishing them from those with isolated AS [15, 24, 25]. As expected, interventricular septal thickness, relative wall thickness, posterior wall thickness, LV mass index, and LA dimensions were found to be significantly higher in patients with AS-CA when compared to those with AS alone [24]. The pathophysiology involved structural infiltration of amyloid fibrils into the aortic valve leaflets, which leads to calcification and fibrosis that eventually worsened the stenosis. Histopathological examination of surgically removed valves in elderly patients confirmed the frequent presence of amyloid deposits, suggesting a direct role in aortic valve disease progression [26].

2.1.1.2 Diagnosis

In clinical practice, there are several clues that should raise suspicion for coexisting CA in a patient with AS. Discrepancies between marked LV wall thickening and low electrocardiogram (ECG) voltages (voltage-to-mass mismatch) are key indicators. Reduced global longitudinal strain (GLS) with apical sparing on echocardiography is another finding suggestive of CA. If these red flags are present, particularly in low-flow low-grade AS cases, physicians should pursue further imaging with CMR or nuclear scintigraphy. Bone scintigraphy using technetium-labeled tracers like 99mTc-DPD has shown high diagnostic accuracy for ATTR amyloidosis, especially with positive Grade 2 or 3 myocardial uptake [17].

2.1.1.3 Management

The management of patients with concomitant AS-CA, particularly ATTR amyloidosis, is complex and requires a tailored, multidisciplinary approach. General heart failure therapies focus on symptom control, with loop diuretics are the mainstay for volume overload. However, these must be used cautiously due to the preload-dependent restrictive pattern of amyloid hearts [27]. Conventional agents such as beta-blockers and calcium channel blockers must be avoided due to their negative chronotropic effect, as they may worsen hypotension or impair cardiac output since those patients are dependent on heart rate in maintaining cardiac output because of the impaired ventricular filling with CA [14, 27]. In cases of acute decompensation, selective therapies like tolvaptan or levosimendan, have shown potential for volume reduction and hemodynamic support [14]. Targeted therapy depends on amyloid subtype. In ATTR amyloidosis, tafamidis is the first-line agent that prevents the formation of final amyloid fibrils. It has demonstrated reduced mortality, decreased cardiovascular hospitalization, and halted the decline in functional capacity and quality of life in clinical trials, though its high cost and limited availability remain challenging [28]. Other emerging therapies, including gene silencers like patisiran and inotersen, offer promising options but require further monitoring and investigation. For AL amyloidosis, chemotherapy targeting plasma cells leading to decrease in light chains formation is the standard of care but carries substantial toxicity, particularly in elderly patients. Aortic valve replacement remains the most definitive management of AS-CA. When it comes to valve replacement, TAVR has emerged as a possibly preferred strategy over surgical aortic valve replacement (SAVR) in patients with AS-CA. Few studies have shown that TAVR is associated with better outcomes, including lower mortality, fewer hospitalizations, and reduced procedural risks when compared to SAVR or medical therapy [27, 29, 30]. However, valve replacement doesn’t always correlate to dramatic clinical improvement, as the underlying myocardial disease remains a significant concern. Certain factors can help predict when valve intervention may offer limited benefit. These include a LVEF under 50%, severely reduced GLS worse than –10%, Grade III diastolic dysfunction, and very low SVi (<30–35 mL/m^2^) [27]. In such cases, patients might be better served by medical management alone and by initiating disease-modifying therapies. Consequently, managing such complicated cases necessitates a multidisciplinary team discussion, involving heart failure, valvular heart disease, and amyloidosis specialists to optimize the management for each patient.

#### 2.1.2 Mitral and Tricuspid Regurgitation (MR & TR)

2.1.2.1 Prevalence of MR/TR in CA©

Valvular regurgitation is common in patients with CA. In a multicenter U.S. study of 345 CA patients (73% male, 110 AL and 235 ATTR), 62% had some degree of MR and 66% had TR on echocardiography​​ [31]. An Italian multi-center registry of 538 CA patients (359 ATTR and 179 AL) found that 55% had at least moderate regurgitation of either the mitral or tricuspid valve: isolated significant MR in 20.8%, isolated significant TR in 12.3%, and combined MR+TR in 22.3% [32]. In that study, the overall prevalence of moderate/severe MR and TR did not differ greatly by CA subtype (ATTR vs AL). Several studies from specialized amyloidosis centers in Europe have reported prevalence rates of significant MR and TR in CA. Chacko *et al*. [33] described a prevalence of ~13% for moderate-or-severe MR and ~17% for moderate-or-severe TR in a cohort of 877 ATTR amyloidosis patients in the UK. Other cohorts have reported intermediate prevalence (~17–24% with at least moderate MR) [34]​​. Another study by Fagot *et al*. [35] reported moderate-or-severe TR in 26.2% of 283 CA patients, with nearly similar frequency in AL (23%) and ATTR (28%) subgroups.

2.1.2.2 Pathophysiology and Mechanisms of Regurgitation in CA

Primary amyloid infiltration of valve leaflets: Amyloid protein (whether light chains or transthyretin) can infiltrate the valve leaflets and chordae tendineae. Autopsy and surgical pathology studies have shown amyloid deposits in mitral and tricuspid valve tissue of CA patients [34]​. This infiltration increases leaflet thickness and stiffness, impairing their mobility and coaptation. A classic study using acoustic microscopy found that mitral valves from CA patients with significant MR were markedly stiffer than those from CA patients with only mild MR or controls​ [36]. In CA patients, echocardiography often reveals valve thickening (especially of the leaflets and subvalvular apparatus) even in the absence of heavy calcification [37]. Notably, unexpected presentation with severe MR due to chordae rupture has been reported in CA patients [38]. Chacko *et al*. [33] observed that in ATTR amyloidosis patients with MR and/or TR, morphological and microscopic abnormalities were present in all different components of the AV apparatus, including the annulus, leaflets, commissures, chordae tendinae, papillary muscles and the surrounding atrial and ventricular myocardium. These were not only due to amyloid deposits but also due to cardiac remodeling [33].

Ventricular remodeling and geometry: CA leads to concentric left ventricular thickening and reduced chamber volumes due to amyloid deposits in the myocardium. This altered ventricular geometry can contribute to MR by restricting the normal systolic motion of the papillary muscles and leaflets [37]. Infiltration of the papillary muscles themselves may also occur [33]. The net effect is a form of functional MR due to impaired leaflet coaptation despite structurally intact leaflets (often classified as Carpentier functional class III dysfunction). Indeed, MR in CA is complex and can fulfill Carpentier classes I, II, or III in different scenarios [34]. For example, leaflet restriction (class III) is common from stiff leaflets or tethering, but occasionally chordae rupture (a class II mechanism, excess motion) has been reported in CA causing acute MR [38]. On the right side, extensive amyloid infiltration of the right ventricle (and interventricular septum) can lead to elevated right-sided filling pressures and functional TR due to ventricular dilation and annular stretching.

Atrial enlargement and functional regurgitation: A hallmark of CA (especially ATTR) is bi-atrial enlargement due to restrictive filling and elevated filling pressures. The consequent dilation of the mitral and tricuspid annuli can produce functional MR or TR in the setting of normal leaflet motion (Carpentier type I). This is further exacerbated by AF (present in a large percentage of CA patients), which leads to loss of atrial contractile function and progressive annular dilation [37, 39]. In fact, the most common mechanism of MR in CA observed in one large study was atrial functional MR, followed by primary infiltrative MR [32]. Likewise, for TR, functional mechanisms secondary to pulmonary hypertension (resulting from left heart failure and amyloid lung congestion) or right atrial enlargement are frequently noted​ [32].

Coexisting degenerative valve disease: Many patients with wtATTR amyloidosis are elderly and may have concomitant age-related valvular degeneration independent of amyloid. For example, fibrocalcific degeneration of the aortic valve (aortic sclerosis/stenosis) frequently coexists with CA [31, 37]. In the mitral valve, annular calcification or fibroelastic degeneration might contribute to MR alongside amyloid. It can be challenging to distinguish pure “amyloid-related” regurgitation from degenerative or functional etiologies in a given patient. In practice, CA patients often have a mixed etiology for MR/TR. For example, an older patient might have mild myxomatous degeneration plus amyloid infiltration plus atrial enlargement, all contributing to regurgitation.

2.1.2.3 Prognosis

Impact of MR on outcomes: Recent data suggest that mitral regurgitation has prognostic importance in CA. Chacko *et al*. [33] followed 877 ATTR amyloidosis patients and found that progression of MR severity over time was one of the strongest predictors of mortality in their cohort. In an earlier analysis (1240 CA patients), moderate-or-severe MR at baseline was associated with worse survival [40]. The finding that worsening MR is a bad sign suggests MR could be a surrogate for disease progression (perhaps reflecting worsening restrictive physiology or AF onset). Another multicenter Italian study provided strong evidence that significant valvular regurgitation portends worse outcomes in CA independent of other factors. Patients were stratified by no significant MR/TR, isolated MR, isolated TR, or combined MR+TR. Those with isolated TR and combined MR/TR had a significantly higher risk of death or heart-failure hospitalization compared to those without significant regurgitation, even after adjusting for age, sex, CA subtype, and cardiac biomarkers [32].

Impact of TR on outcomes: TR in CA is emerging as an important prognostic marker. In the multicenter Italian study, authors found that isolated TR carried the highest hazard for adverse outcomes​; even higher than those with combined MR/TR (adjusted hazard ratio for all-cause death/HF hospitalization was 2.75 for isolated TR and 2.31 for combined MR/TR) [32]. Similarly, in a U.S. cohort of 345 CA patients, moderate/severe TR was associated with significantly worse median survival (2.3 years) versus none/mild TR (3.35 years) [31]. In that study, TR even had a clearer association with mortality than MR [31]. One explanation could be that severe TR reflects advanced right-sided failure and diastolic dysfunction, which in CA indicates a more advanced disease. Additionally, TR aggravates renal and hepatic congestion, potentially accelerating multi-organ failure in those patients.

2.1.2.4 Management

The management of CA with MR and TR requires a two-pronged approach: treating the underlying amyloid disease to halt or slow progression and addressing the valvular dysfunction to relieve symptoms and improve hemodynamics. Amyloid-targeted therapy includes chemotherapy for AL amyloidosis and transthyretin stabilizing agents such as tafamidis for ATTR amyloidosis. Supportive HF therapy is generally required with the similar limitations discussed in AS section. Maintaining sinus rhythm or at least controlled rate in AF is crucial to maximize cardiac output and minimize regurgitation volumes. Amiodarone is often the drug of choice for AF in CA since rate control agents are usually intolerable in this population. Transcatheter Edge-to-Edge Repair (TEER, MitraClip) has emerged as an attractive option to treat MR. A German case series of 5 amyloidosis patients (4 ATTR, 1 AL) who underwent MitraClip reported 100% procedural success with durable MR reduction [41]. They even suggested a survival benefit compared to matched CA patients with severe MR who did not get TEER. A larger study of 120 TEER patients included 23 with CA. They achieved 100% acute procedural success and significant MR reduction in all CA cases [39]. However, patients with dual CA and MR had worse outcomes (HF hospitalization/all-cause mortality) compared to those with MR alone.

Transcatheter therapies for TR have lagged behind MitraClip but are now emerging. The PASCAL-Ace and the TriClip-Systems have been used for the transcatheter tricuspid valve repair. The largest case series to date, from the University Heidelberg, treated 8 ATTR amyloidosis patients (and 21 non-amyloid controls) with the PASCAL device for severe TR [42]. The procedural success was 100%, and at 3 months follow-up, the TR grade improved significantly and NYHA functional class improved. Importantly, when comparing the outcomes of the CA patients to non-CA TR patients, there was no significant difference in survival or heart failure hospitalization at short-term follow-up [42]. This suggests that CA patients can benefit from TR reduction similarly to other patients, at least in terms of symptomatic relief, without excessive peri-procedural risk.

#### 2.1.3 Mitral and Tricuspid Stenosis

Unlike the high prevalence of AS in CA patients, MS and TS seem to be extremely rare in this patient population. Data from large CA centers did not show any clinically relevant proportion of either MS or TS [34]. When carefully searching the literature, only very few case reports were found to report MS or TS in this subset of patients [43, 44, 45]. This can be attributed to the fact that amyloid deposition in the valve apparatus often causes annular dilatation, increasing leaflet thickness and stiffness which can counteract the potential for stenotic processes, making stenosis less common.

### 2.2 Arrhythmias and Electrophysiology

#### 2.2.1 Atrial Fibrillation

2.2.1.1 Epidemiology and Prevalence

CA is a restrictive cardiomyopathy that results from the extracellular deposition of misfolded proteins, and its clinical recognition has expanded rapidly in recent years. One shared challenge across the spectrum of CA is the high burden of cardiac dysrhythmias, especially AF. In patients with wtATTR, AF is remarkably common, occurring in nearly 70% of cases, as shown by Mints *et al*. [46]. This is a striking contrast to the AF prevalence observed in other forms of heart failure, where it tends to range from 13% to 27% [47]. In their cohort of 146 biopsy-confirmed wtATTR patients, AF was associated with more severe diastolic dysfunction but did not confer an independent mortality risk [46]. This finding contrasts with non-amyloid heart failure cohorts, where AF is consistently associated with worse prognosis [48]. Other studies reported a 26% prevalence of AF among those with AL amyloid with an overall prevalence of 44% in CA patients [49]. The disproportionately high burden of AF is thought to be multifactorial including direct amyloid fibril infiltration of the myocardium leading to fibrosis, cardiac remodeling, and electromechanical dissociation. Additionally, the restrictive pattern seen in CA leads to elevated filling pressure and atrial dilatation which is arrhythmogenic [49].

2.2.1.2 Role of Anticoagulation

As for anticoagulation strategy, Mints *et al*. [46] found that warfarin was used in 78% of patients, while direct oral anticoagulants (DOACs) were used in only 17%. Generally, there is insufficient data comparing the use of DOACs vs warfarin in CA patients; hence, the anticoagulation choice currently following general guidelines for AF [50]. Safety and efficacy of DOACs in the general AF population may support their use in ATTR amyloidosis patients without contraindications. However, renal and gastrointestinal involvement in AL amyloidosis may favor the use of warfarin [51, 52, 53]. Left atrial appendage (LAA) closure may still be considered for patients with contraindications to anticoagulant therapy or those who developed complications [54].

2.2.1.3 Role of Rate Control

Beyond anticoagulation, rate and rhythm control in CA present unique challenges. Amyloid infiltration of the myocardium can cause sinus node dysfunction and AV block, leading to chronotropic incompetence. This complicates the use of beta-blockers, calcium channel blockers (CCBs), and digoxin, which may further reduce cardiac output in patients who already rely on higher resting heart rates to maintain perfusion due to the restrictive nature of CA. For instance, CCBs can bind to myocardial amyloid fibrils, potentially leading to enhanced chronotropic effect [55]. One the other hand, Rubinow *et al*. [56] found that digoxin can bind avidly to amyloid fibrils *in vitro*; hence, increasing the risk of potential toxicity. Using these agents was reported to worsen the outcomes due to precipitation of heart failure, hypotension, and possibly cardiogenic shock [46, 49, 54]. In line with the current evidence, Mints *et al*. [46] found a trend towards worsening survival outcomes in ATTR amyloidosis patients with AF who have been managed with rate control vs rhythm control strategy; though, the difference did not reach the statistical significance (HR 1.70; *p* = 0.08).

2.2.1.4 Role of Rhythm Control

Given the drawbacks and associated risks of rate control strategy in CA patients, many experts may prefer the rhythm control strategy in this population [49]. On one hand, it can help to avoid the use of rate control agents. On the other hand, the use of antiarrhythmic agents seems to be well tolerated in those patients and provides significant symptomatic improvement [54]. In Mints *et al*.’s study [46], about one-third of ATTR amyloidosis patients were treated with rhythm control strategies, and amiodarone was the most commonly used agent due to its tolerability and effectiveness. It’s noteworthy that from a physiological point of view, the benefit of rhythm control may be questionable in asymptomatic patients with restrictive physiology, as ventricular filling occurs mainly during early diastole with only negligible effect of atrial contraction [49]. This highlights the need for further investigation to decide whether rhythm control strategy should be prioritized in this patient population.

2.2.1.5 Role of Catheter Ablation

Data about the safety and efficacy of catheter ablation in CA patients with AF are limited. Several small-size studies have reported high recurrence rates of AF in patients with CA who have been received catheter ablation [57, 58, 59]. Nonetheless, Donnellan *et al*. [59] reported that ATTR amyloidosis patients who underwent ablation had lower hospitalizations and mortality rates when compared to a matched control group of medically managed patients. Given the available data, catheter ablation may be a reasonable option in patients who have contraindications to rate or rhythm control medications or those who have refractory AF despite medical management [54].

2.2.1.6 Role of Screening

Given the advanced age of ATTR amyloidosis patients, high prevalence of AF, and the importance of early detection in improving patient outcomes, arrhythmia screening may be warranted. Subclinical AF is common and often detected only via implantable devices. Routine ECGs and Holter monitoring should be part of standard follow-up, especially in older adults with unexplained heart failure, thickened ventricular walls, or low voltage on ECG. For instance, Routine ambulatory heart rhythm monitoring has been shown to be effective in detecting subclinical AF/AFL in patients with ATTR amyloidosis, which can lead to timely anticoagulation and potentially reduce the risk of stroke [60]. In a study by Dale *et al*. [60], about half of the patients with new onset AF were discovered incidentally on ambulatory monitoring. Ambulatory monitoring subsequently led to starting anticoagulation in 82% of these patients with newly incident AF, and none had a thromboembolic event during follow up [60]. Taken into consideration that anticoagulation is recommended for AF in CA patients irrespective of the CHADVASc score due to the high risk of thromboembolic events, early detection is crucial to allow for timely initiation of anticoagulation and rhythm control, which may decrease the risk of stroke and improve clinical outcomes.

## 3. CA in Clinical Practice: Navigating the Gray Zones and Phenotypic Challenges

### 3.1 Thromboembolic and Hemorrhagic Risks in CA: A Delicate Balance

CA is associated with a unique and paradoxical hemostatic profile that increases the risk of both thromboembolic and bleeding complications, as shown in Table 4. While AF is highly prevalent, especially in ATTR amyloidosis (~50%), thromboembolic events in CA extend beyond the presence of arrhythmia. Stroke, systemic embolism, and intracardiac thrombus formation have been reported even in patients with sinus rhythm, likely due to atrial mechanical dysfunction, electromechanical dissociation, endothelial damage, and systemic hypercoagulability [49, 61].

**Table 4.** **Contrasting pathophysiologic mechanisms of bleeding and thromboembolism in cardiac amyloidosis (CA)**.

Bleeding Mechanisms	Thromboembolic Mechanisms
Amyloid angiopathy leads to vascular fragility and spontaneous bleeding	Atrial electromechanical dissociation causes blood stasis despite sinus rhythm
Capillary fragility, especially in mucosal and periorbital regions	Atrial fibrillation promotes left atrial appendage thrombus formation
Factor X deficiency in AL amyloidosis due to hepatic sequestration or amyloid binding	Dysfunction of the left atrial appendage reduces blood flow velocity and promotes thrombus
Excessive fibrinolysis due to upregulated uPA expression in AL	Atrial dilation and fibrosis support thrombogenesis even without AF
Amyloid infiltration of the GI tract increases mucosal bleeding risk	Nephrotic syndrome in AL causes loss of antithrombin and protein S, increasing coagulability
Anticoagulation-induced bleeding, especially with VKAs	Ventricular dysfunction and low cardiac output lead to intracardiac stasis
Bleeding after minor trauma due to orthostatic falls from autonomic neuropathy	Atrial standstill results in total atrial dysfunction and high thrombotic risk even in sinus rhythm
Clinical bleeding with near-normal coagulation parameters (AL-specific defect)	Light chain-induced endothelial inflammation increases thrombotic risk

VKA, vitamin K antagonist.

Atrial amyloid infiltration may result in atrial standstill, contributing to stasis in the LAA. Importantly, thrombi have been detected in the LAA via transesophageal echocardiography (TEE) even in patients without AF or with CHA₂DS₂-VASc scores that would not otherwise warrant anticoagulation [62]. Donnellan *et al*. [62] found that 88% of CA patients with LAA thrombi were therapeutically anticoagulated for at least three weeks prior to TEE, while another study showed 46% of thrombi were identified despite guideline-based anticoagulation or a recent-onset arrhythmia [62, 63]. These findings highlight the inadequacy of conventional stroke risk stratification tools in this population.

As a result, expert consensus suggests a lower threshold for anticoagulation in CA, including patients in sinus rhythm with evidence of atrial mechanical dysfunction [50, 54]. Furthermore, TEE prior to cardioversion should be strongly considered regardless of rhythm duration or anticoagulation status [62].

Despite the prothrombotic risk, bleeding complications are also a major concern in CA. Amyloid deposition in the vasculature renders vessel walls fragile, increasing the risk of spontaneous bleeding, petechiae, and pathognomonic findings such as periocular purpura in AL amyloidosis [64, 65]. In addition to physical disruption, amyloid fibrils, particularly in AL, may bind and inactivate clotting factors, most notably Factor X, leading to acquired coagulopathies [66, 67]. Cases of severe hyperfibrinolysis with hypofibrinogenemia have also been reported, which may respond to protease inhibition [64, 68].

This dual risk creates significant therapeutic dilemmas. Although anticoagulation is empirically beneficial, reducing stroke and systemic embolism even in high-risk CA patients (odds ratio for embolism with anticoagulation ~0.09), the risk of major hemorrhage must be managed carefully [49]. Direct oral anticoagulants (DOACs) have shown promise in this setting, offering better stroke prevention to warfarin without an increase in bleeding risk. Two meta-analyses of CA patients treated with DOACs demonstrated fewer thromboembolic events with no increase in bleeding complications compared to vitamin K antagonists [53, 69]. This may be attributed to the predictable pharmacodynamics of DOACs and the avoidance of warfarin-related vascular calcification, which could worsen amyloid angiopathy.

In AL amyloidosis, addressing the underlying plasma cell dyscrasia through chemo-immunotherapy (e.g., proteasome inhibitors, anti-CD38 antibodies) can reduce both thrombotic and hemorrhagic risks by mitigating the production of pathogenic light chains [70, 71]. Therefore, early hematologic control remains central to management.

### 3.2 Amyloid Heart vs HFpEF 

The result of cardiac amyloid deposition is a stiff, restrictive cardiomyopathy. The ventricles fill poorly despite preserved ejection fraction, mimicking HFpEF in symptoms and hemodynamics [72]. Patients typically present with exercise intolerance, dyspnea, and edema, a clinical picture nearly indistinguishable from ordinary HFpEF [73]. Indeed, wild-type ATTR amyloidosis is now understood to be an underappreciated cause of HFpEF in the elderly; post-mortem studies reveal clinically silent ATTR deposits in ~25% of individuals over 85, and observational cohorts have confirmed ATTR amyloidosis as an etiologic subset of HFpEF in older patients [74, 75]. This overlap means CA often goes unrecognized, erroneously ascribed to “garden-variety” hypertensive HFpEF.

However, several features distinguish amyloid cardiomyopathy on careful evaluation. Amyloid infiltration typically produces a disproportionate increase in ventricular wall thickness (often ≥12–15 mm) that, unlike true hypertrophy, paradoxically accompanies low QRS voltages on ECG due to electrical insulation by amyloid, a red flag for infiltrative disease [76]. CA also frequently involves the atria and conduction system, causing AF, atrioventricular block, and even atrial standstill, whereas primary HFpEF more often has preserved sinus node function until late [77, 78]. Patients with CA may have extracardiac signs (“amyloid red flags”) such as carpal tunnel syndrome, peripheral neuropathy, biceps tendon rupture, or periorbital purpura, which are absent in routine HFpEF [79]. Recognizing these clues is vital and clinicians are urged to screen for CA in any HFpEF patient with atypical features, as prompt diagnosis permits timely disease-modifying therapy.

In practice, a combination of advanced imaging (strain echocardiography showing apical sparing pattern, technetium-PYP nuclear scintigraphy for ATTR amyloidosis, and cardiac MRI with global late gadolinium enhancement), plus tissue biopsy or genetic testing, is used to confirm the amyloid etiology in suspected cases. The stakes of early detection are high as untreated CA has a median survival often just 2–3 years once heart failure is overt, and late diagnosis is a major cause of shortened survival [5, 18].

### 3.3 Microvascular Dysfunction: Amyloid’s “Invisible” Ischemia

A key pathophysiologic intersection between CA and HFpEF lies in the microvasculature. Both diseases feature microvascular dysfunction, but the underlying mechanisms differ markedly. In HFpEF without amyloid, comorbidities (hypertension, diabetes, obesity, etc.) drive systemic inflammation and endothelial dysfunction, leading to decreased nitric oxide bioavailability and a cascade of cGMP-PKG pathway impairment [80, 81, 82]. This in turn causes titin stiffening, diffuse fibrosis, and capillary rarefaction—a process that chokes off blood supply in the myocardium. Amyloid cardiomyopathy exhibits greater microvascular impairment, but via direct encroachment and toxicity rather than primarily endothelial inflammation. Amyloid fibrils deposit within intramyocardial arterioles and capillary walls, distorting the architecture and even obliterating small vessels [83, 84].

Histologic studies in CA reveal severe capillary rarefaction and obstruction of intramural coronary arteries by amyloid, whereas interstitial fibrosis is relatively mild. Essentially, amyloid replaces what fibrosis and hypertrophy occupy in other cardiomyopathies [85, 86]. In AL amyloidosis, toxic light chains further injure the endothelium and smooth muscle via oxidative stress, compounding the reduction in coronary flow reserve [87]. The net effect is profound microvascular dysfunction and “supply-demand” mismatch in the heart. Patients with CA frequently experience angina or exercise-induced ischemia despite completely normal epicardial coronaries [80, 88].

### 3.4 Managing the Asymptomatic and the “Pre-CA” Patient

The paradigm of “early intervention” in CA extends to those who have not yet developed overt cardiac symptoms. Thanks to greater awareness and familial screening, clinicians are increasingly encountering individuals who are genotype-positive for hereditary ATTR amyloidosis (ATTRv) or have evidence of amyloid deposits but minimal clinical manifestations [89]. These scenarios pose challenging questions: when should therapy begin in a condition that is ultimately fatal if left to declare itself? In hereditary ATTR amyloidosis, once a pathogenic TTR mutation (e.g., Val122Ile or Thr60Ala) is identified in a patient or relative, guidelines recommend periodic surveillance for disease onset. This typically includes annual or semiannual evaluations with cardiac biomarkers (troponin, NT-proBNP), ECG and echocardiography with strain imaging, and perhaps radionuclide scanning, to detect the earliest cardiac involvement [90, 91].

The goal is to catch the transition from an asymptomatic carrier (Stage 0 ATTR amyloidosis) to early CA (Stage 1) as soon as it occurs. At that inflection point, emerging amyloid deposition with maybe mild thickening or subtle symptom onset, initiating therapy could theoretically slow or prevent progression [92]. While no trial has yet tested prophylactic treatment in truly asymptomatic ATTR mutation carriers, many experts advocate a low threshold to start a TTR stabilizer once there are objective signs of cardiac involvement, even if heart failure symptoms are absent. This proactive approach is supported by the observation that patients derive far greater benefit from therapy when started in earlier stages of disease [90].

In the pivotal ATTR-ACT trial, tafamidis given to ATTR cardiomyopathy patients led to a 30% relative reduction in mortality and lower heart failure hospitalizations; notably, these benefits were most pronounced in those with mild (NYHA class I–II) symptoms, whereas patients in advanced (class III) heart failure showed no net hospitalization benefit and had worse outcomes if treated late. Early treatment not only prolongs survival but preserves quality of life and functional capacity to a far greater extent than salvage therapy in advanced CA [28].

For asymptomatic mutation carriers, management currently focuses on close monitoring and timely intervention at the first sign of organ involvement. Some propose that TTR stabilizers (like tafamidis or upcoming agents such as acoramidis) could be started in a “stage 0.5” scenario—say, a genotype-positive 70-year-old with mild increased wall thickness and abnormal strain but no heart failure symptoms—to delay the onset of overt cardiomyopathy [93]. Such an approach must be weighed against cost and uncertain long-term benefit, but ongoing studies and real-world experience will inform the threshold for prophylactic therapy.

Similarly, in AL amyloidosis, patients with an indolent plasma cell clone (MGUS or smoldering myeloma) and low-level light chain production might be observed until biomarkers or imaging suggest early cardiac infiltration, at which point definitive therapy (chemotherapy and stem-cell transplant if eligible) is instituted before fulminant AL-CM ensues [94]. The overarching principle is that CA, once symptomatic, portends irreversible organ damage, so intercepting the disease earlier can dramatically alter the patient’s trajectory. This principle is driving efforts to develop sensitive screening tools (e.g., amyloid-specific PET tracers, advanced echocardiographic strain and electrocardiogram algorithms) to detect “amyloid in the wild” well before heart failure develops [95, 96]. It also raises ethical and logistical considerations: how to surveil at-risk individuals longitudinally, how to communicate risk, and when the benefits of early therapy outweigh the risks and costs. Multidisciplinary amyloidosis centers now often convene genotype-positive clinics for mutation carriers, where patients receive education, baseline testing, and a tailored follow-up plan [97]. This approach will likely become standard as our ability to identify preclinical CA grows.

### 3.5 Common Clinical Scenarios of Incidental CA

CA often presents with nonspecific cardiovascular symptoms and is frequently misdiagnosed as more common conditions until further diagnostic evaluation is prompted by unresponsiveness to standard therapies. A classic clinical scenario involves patients who present with signs and symptoms of heart failure—such as exertional dyspnea, fatigue, peripheral edema, and fluid retention—yet demonstrate poor or absent response to guideline-directed medical therapy. This lack of improvement typically prompts advanced cardiac evaluation, which may reveal infiltrative cardiomyopathy due to CA [13]. Another common presentation is exertional chest pain in the context of normal coronary angiography, initially managed as microvascular angina or Syndrome X. Persistence of symptoms despite treatment, especially when accompanied by restrictive features on echocardiography or cardiac magnetic resonance imaging (MRI), may raise suspicion for amyloid infiltration. Such cases underscore the importance of considering CA in the differential diagnosis of angina with non-obstructive coronary arteries [98, 99, 100, 101]. Arrhythmias and conduction system abnormalities are also common initial manifestations. Patients may present with AF, bradyarrhythmias, or varying degrees of heart block that are refractory to conventional therapies. Progression of conduction disturbances, especially in the absence of structural abnormalities commonly associated with such rhythms, should prompt consideration of CA as an underlying etiology [102, 103].

Additionally, patients with increased left ventricular (LV) wall thickness on echocardiography are frequently misdiagnosed with hypertrophic cardiomyopathy (HCM) or hypertensive heart disease. However, a discrepancy between marked LV hypertrophy and low QRS voltages on ECG, as well as poor response to treatment, should alert clinicians to the possibility of amyloid cardiomyopathy [104].

In patients with longstanding hypertension who present with heart failure symptoms and echocardiographic evidence of LV hypertrophy, CA should be considered when there is a suboptimal response to optimized antihypertensive and heart failure therapies. The presence of low ECG voltages, a restrictive filling pattern on Doppler imaging, or disproportionate wall thickening further supports this suspicion [76, 105].

Extra-cardiac manifestations may also serve as early indicators of amyloidosis. A history of bilateral carpal tunnel syndrome, lumbar spinal stenosis, or spontaneous biceps tendon rupture, particularly in elderly individuals, should prompt evaluation for transthyretin amyloidosis, especially when cardiac symptoms emerge later in the disease course [106, 107].

Finally, in patients diagnosed with HFpEF who fail to improve with conventional therapies, further evaluation for infiltrative diseases is warranted. In this context, CA should be suspected, and diagnostic confirmation can often be achieved using bone scintigraphy (e.g., 99mTc-PYP) or endomyocardial biopsy [108, 109].

### 3.6 Financial Toxicity and Cost-related Challenges

The treatment of ATTR CA has advanced significantly with the approval of tafamidis in 2019, the first pharmacologic agent shown to reduce all-cause mortality and cardiovascular hospitalizations in this population. Despite its clinical benefits, tafamidis entered the market with a prohibitive list price of approximately $225,000 per patient per year, making it one of the most expensive cardiovascular drugs ever approved. This high cost has raised substantial concerns regarding financial toxicity, cost-effectiveness, and equitable access to care.

Health economic evaluations have demonstrated that tafamidis, at its current price, fails to meet standard cost-effectiveness thresholds. A cost-effectiveness analysis estimated that the drug’s annual cost would need to be reduced by more than 90%, to about $16,000, to be considered cost-effective based on a $100,000 per quality-adjusted life year (QALY) threshold. The burden of this cost is particularly impactful in the elderly population typically affected by ATTR amyloidosis, many of whom rely on Medicare or fixed incomes. As a result, patients often face high out-of-pocket expenses and may be forced to choose between accessing life-prolonging therapy and maintaining financial stability [110].

In real-world practice, the high cost of tafamidis has led to insurance-related delays, denials, and treatment discontinuation due to unaffordable copayments. These access barriers risk exacerbating healthcare disparities unless mitigated by policy reforms or pricing adjustments. From a system-wide perspective, this financial challenge underscores the importance of early diagnosis and intervention. Clinical and economic models indicate that initiating tafamidis in early-stage ATTR amyloidosis (Stage I or II) improves both survival and the cost-effectiveness of screening and therapy by delaying heart failure progression and reducing hospitalizations. Conversely, late-stage use yields limited benefit and poor value, particularly in the setting of irreversible myocardial damage [110, 111, 112].

Future cost-containment strategies may include broader adoption of lower-cost alternatives such as diflunisal or acoramidis, and policy solutions like Medicare drug price negotiations or value-based pricing models. Until such measures are implemented, clinicians must advocate for early identification of eligible patients and judicious use of high-cost therapies to maximize clinical benefit while mitigating financial harm.

## 4. Conclusion

CA presents with protean clinical features that mimic or overlap with more familiar entities like HFpEF, aortic stenosis, and age-related conduction disorders. This phenotypic camouflage often delays diagnosis and impairs timely intervention. However, the growing availability of advanced imaging, molecular diagnostics, and subtype-specific therapies has made early and accurate identification more feasible and more impactful than ever before.

This review has highlighted key aspects of CA in real-world practice, from red flag signs and microvascular dysfunction to valvular involvement and arrhythmic complications. It has emphasized that amyloid deposition does not merely stiffen the myocardium but also alters atrial and valvular function, complicating hemodynamics and therapeutic choices. The high incidence of AF and thromboembolic events, sometimes even in sinus rhythm, underscores the inadequacy of traditional risk scores in this population and calls for a tailored anticoagulation strategy.

Staging systems now inform prognosis and therapy selection, yet they remain imperfect, particularly when extrapolated across amyloid subtypes. Similarly, therapeutic breakthroughs like tafamidis have reshaped management, but their cost poses barriers that reinforce the urgency of early-stage treatment and careful resource allocation. Screening of high-risk groups, including those with carpal tunnel syndrome, aortic stenosis, or plasma cell disorders, offers a pathway to detect CA before irreversible organ damage occurs.

Ultimately, effective management of CA requires a multidisciplinary approach grounded in early suspicion, accurate diagnosis, and equitable access to therapy. As the field evolves, this alignment of clinical vigilance, targeted intervention, and health system integration holds the promise of significantly improved outcomes.

## 5. Limitations

This study has a few important limitations. Much of the evidence referenced is based on retrospective or observational data, limiting causal interpretation. The heterogeneity of CA, particularly between AL and ATTR amyloidosis subtypes, complicates the application of uniform screening or staging strategies. Additionally, while we incorporated up-to-date literature, emerging diagnostics or therapies may not be fully addressed.

Finally, AI tools including ChatGPT-4o (OpenAI), QuillBot, Grammarly, and EndNote were used to support grammar and structure, but all scientific interpretations and content were independently written and reviewed by the authors, who take full responsibility for the manuscript’s accuracy and integrity.

## References

[1] Xin Y, Hu W, Chen X, Hu J, Sun Y, Zhao Y (2019). Prognostic impact of light-chain and transthyretin-related categories in cardiac amyloidosis: A systematic review and meta-analysis. *Hellenic Journal of Cardiology: HJC = Hellenike Kardiologike Epitheorese*.

[2] Fontana M, Ioannou A, Cuddy S, Dorbala S, Masri A, Moon JC (2025). The Last Decade in Cardiac Amyloidosis: Advances in Understanding Pathophysiology, Diagnosis and Quantification, Prognosis, Treatment Strategies, and Monitoring Response. *JACC. Cardiovascular Imaging*.

[3] Yamamoto H, Yokochi T (2019). Transthyretin cardiac amyloidosis: an update on diagnosis and treatment. *ESC Heart Failure*.

[4] Merlini G, Dispenzieri A, Sanchorawala V, Schönland SO, Palladini G, Hawkins PN (2018). Systemic immunoglobulin light chain amyloidosis. *Nature Reviews. Disease Primers*.

[5] Grogan M, Scott CG, Kyle RA, Zeldenrust SR, Gertz MA, Lin G (2016). Natural History of Wild-Type Transthyretin Cardiac Amyloidosis and Risk Stratification Using a Novel Staging System. *Journal of the American College of Cardiology*.

[6] Gillmore JD, Damy T, Fontana M, Hutchinson M, Lachmann HJ, Martinez-Naharro A (2018). A new staging system for cardiac transthyretin amyloidosis. *European Heart Journal*.

[7] Cheng RK, Levy WC, Vasbinder A, Teruya S, De Los Santos J, Leedy D (2020). Diuretic Dose and NYHA Functional Class Are Independent Predictors of Mortality in Patients With Transthyretin Cardiac Amyloidosis. *JACC. CardioOncology*.

[8] Grasso M, Cavaliere C, Vilardo V, Tagliani M, Di Toro A, Urtis M (2025). Present and future of endomyocardial biopsy in cardiac amyloidosis. *European Heart Journal Supplements: Journal of the European Society of Cardiology*.

[9] Anderson L, Pennell D (2008). The role of endomyocardial biopsy in the management of cardiovascular disease: a scientific statement from the American Heart Association, the American College of Cardiology, and the European Society of Cardiology. *European Heart Journal*.

[10] Dorbala S (2023). Expanding indications for non-biopsy diagnosis of transthyretin amyloid cardiomyopathy. *European Heart Journal*.

[11] Briasoulis A, Bampatsias D, Papamichail A, Kuno T, Skoularigis J, Xanthopoulos A (2023). Invasive and Non-Invasive Diagnostic Pathways in the Diagnosis of Cardiac Amyloidosis. *Journal of Cardiovascular Development and Disease*.

[12] Westin O, Fosbøl EL, Maurer MS, Leicht BP, Hasbak P, Mylin AK (2022). Screening for Cardiac Amyloidosis 5 to 15 Years After Surgery for Bilateral Carpal Tunnel Syndrome. *Journal of the American College of Cardiology*.

[13] González-López E, Gallego-Delgado M, Guzzo-Merello G, de Haro-Del Moral FJ, Cobo-Marcos M, Robles C (2015). Wild-type transthyretin amyloidosis as a cause of heart failure with preserved ejection fraction. *European Heart Journal*.

[14] Jaiswal V, Agrawal V, Khulbe Y, Hanif M, Huang H, Hameed M (2023). Cardiac amyloidosis and aortic stenosis: a state-of-the-art review. *European Heart Journal Open*.

[15] Nitsche C, Scully PR, Patel KP, Kammerlander AA, Koschutnik M, Dona C (2021). Prevalence and Outcomes of Concomitant Aortic Stenosis and Cardiac Amyloidosis. *Journal of the American College of Cardiology*.

[16] Caponetti AG, Accietto A, Saturi G, Ponziani A, Sguazzotti M, Massa P (2023). Screening approaches to cardiac amyloidosis in different clinical settings: Current practice and future perspectives. *Frontiers in Cardiovascular Medicine*.

[17] Gillmore JD, Maurer MS, Falk RH, Merlini G, Damy T, Dispenzieri A (2016). Nonbiopsy Diagnosis of Cardiac Transthyretin Amyloidosis. *Circulation*.

[18] Maurer MS, Elliott P, Comenzo R, Semigran M, Rapezzi C (2017). Addressing Common Questions Encountered in the Diagnosis and Management of Cardiac Amyloidosis. *Circulation*.

[19] Maggialetti N, Torrente A, Lorusso G, Villanova I, Ficco M, Gravina M (2024). Role of Cardiovascular Magnetic Resonance in Cardiac Amyloidosis: A Narrative Review. *Journal of Personalized Medicine*.

[20] Müller ML, Spethmann S, Messroghli D, Brand A, Mattig I, Hahn K (2025). Accuracy of Established Prognostic Staging Systems for Cardiac Transthyretin Amyloidosis in the Tafamidis Era. *JACC. Advances*.

[21] Garcia-Pavia P, Bengel F, Brito D, Damy T, Duca F, Dorbala S (2021). Expert consensus on the monitoring of transthyretin amyloid cardiomyopathy. *European Journal of Heart Failure*.

[22] De Andrés-Cardelle F, Barge-Caballero G, López-Pérez M, López-López A, González-Babarro E, Gutiérrez-Feijoo M (2025). Usefulness of the Columbia score for predicting outcomes in patients with transthyretin amyloid cardiomyopathy. Analysis of the Galician registry of cardiac amyloidosis. *Amyloid: the International Journal of Experimental and Clinical Investigation: the Official Journal of the International Society of Amyloidosis*.

[23] Castaño A, Narotsky DL, Hamid N, Khalique OK, Morgenstern R, DeLuca A (2017). Unveiling transthyretin cardiac amyloidosis and its predictors among elderly patients with severe aortic stenosis undergoing transcatheter aortic valve replacement. *European Heart Journal*.

[24] Jaiswal V, Ang SP, Chia JE, Abdelazem EM, Jaiswal A, Biswas M (2022). Echocardiographic predictors of presence of cardiac amyloidosis in aortic stenosis. *European Heart Journal. Cardiovascular Imaging*.

[25] Cavalcante JL, Rijal S, Abdelkarim I, Althouse AD, Sharbaugh MS, Fridman Y (2017). Cardiac amyloidosis is prevalent in older patients with aortic stenosis and carries worse prognosis. *Journal of Cardiovascular Magnetic Resonance: Official Journal of the Society for Cardiovascular Magnetic Resonance*.

[26] Kristen AV, Schnabel PA, Winter B, Helmke BM, Longerich T, Hardt S (2010). High prevalence of amyloid in 150 surgically removed heart valves–a comparison of histological and clinical data reveals a correlation to atheroinflammatory conditions. *Cardiovascular Pathology: the Official Journal of the Society for Cardiovascular Pathology*.

[27] Ternacle J, Krapf L, Mohty D, Magne J, Nguyen A, Galat A (2019). Aortic Stenosis and Cardiac Amyloidosis: JACC Review Topic of the Week. *Journal of the American College of Cardiology*.

[28] Maurer MS, Schwartz JH, Gundapaneni B, Elliott PM, Merlini G, Waddington-Cruz M (2018). Tafamidis Treatment for Patients with Transthyretin Amyloid Cardiomyopathy. *The New England Journal of Medicine*.

[29] Galat A, Guellich A, Bodez D, Slama M, Dijos M, Zeitoun DM (2016). Aortic stenosis and transthyretin cardiac amyloidosis: the chicken or the egg?. *European Heart Journal*.

[30] Cannata F, Chiarito M, Pinto G, Villaschi A, Sanz-Sánchez J, Fazzari F (2022). Transcatheter aortic valve replacement in aortic stenosis and cardiac amyloidosis: a systematic review and meta-analysis. *ESC Heart Failure*.

[31] Minga I, Kwak E, Hussain K, Wathen L, Gaznabi S, Singh L (2024). Prevalence of valvular heart disease in cardiac amyloidosis and impact on survival. *Current Problems in Cardiology*.

[32] Tomasoni D, Aimo A, Porcari A, Bonfioli GB, Castiglione V, Saro R (2024). Prevalence and clinical outcomes of isolated or combined moderate to severe mitral and tricuspid regurgitation in patients with cardiac amyloidosis. *European Heart Journal. Cardiovascular Imaging*.

[33] Chacko L, Karia N, Venneri L, Bandera F, Passo BD, Buonamici L (2022). Progression of echocardiographic parameters and prognosis in transthyretin cardiac amyloidosis. *European Journal of Heart Failure*.

[34] Duca F, Kronberger C, Willixhofer R, Bartko PE, Bergler-Klein J, Nitsche C (2023). Cardiac Amyloidosis and Valvular Heart Disease. *Journal of Clinical Medicine*.

[35] Fagot J, Lavie-Badie Y, Blanchard V, Fournier P, Galinier M, Carrié D (2021). Impact of tricuspid regurgitation on survival in patients with cardiac amyloidosis. *ESC Heart Failure*.

[36] Masugata H, Mizushige K, Senda S, Kinoshita A, Nozaki S, Matsuo H (2000). Physical properties of the mitral valve tissue assessed by tissue sound speed in cardiac amyloidosis: relationship to the severity of mitral regurgitation. *Ultrasound in Medicine & Biology*.

[37] Aimo A, Camerini L, Fabiani I, Morfino P, Panichella G, Barison A (2024). Valvular heart disease in patients with cardiac amyloidosis. *Heart Failure Reviews*.

[38] Nishi H, Mitsuno M, Ryomoto M, Miyamoto Y (2008). Severe mitral regurgitation due to cardiac amyloidosis–a rare reason for ruptured chordae. *Interactive Cardiovascular and Thoracic Surgery*.

[39] Donà C, Nitsche C, Koschutnik M, Heitzinger G, Mascherbauer K, Kammerlander AA (2022). Unveiling Cardiac Amyloidosis, its Characteristics, and Outcomes Among Patients With MR Undergoing Transcatheter Edge-to-Edge MV Repair. *JACC. Cardiovascular Interventions*.

[40] Chacko L, Martone R, Bandera F, Lane T, Martinez-Naharro A, Boldrini M (2020). Echocardiographic phenotype and prognosis in transthyretin cardiac amyloidosis. *European Heart Journal*.

[41] Volz MJ, Pleger ST, Weber A, Geis NA, Hamed S, Mereles D (2021). Initial experience with percutaneous mitral valve repair in patients with cardiac amyloidosis. *European Journal of Clinical Investigation*.

[42] Hoerbrand IA, Volz MJ, Aus dem Siepen F, Aurich M, Schlegel P, Geis NA (2023). Initial experience with transcatheter tricuspid valve repair in patients with cardiac amyloidosis. *ESC Heart Failure*.

[43] Kwak S, Lim S, Kim YJ, Kim HK (2024). Isolated severe mitral stenosis as an uncommon manifestation of cardiac amyloidosis. *Journal of Cardiovascular Imaging*.

[44] Randhawa VK, Vakamudi S, Phelan DM, Samaras CJ, McKenney JK, Hanna M (2020). Mitral and tricuspid stenosis caused by light chain cardiac amyloid deposition. *ESC Heart Failure*.

[45] Frumkin D, Taube ET, Stangl K, Knebel F (2019). Rapid progression of aortic and mitral stenosis in a patient with AA amyloidosis: a case report. *European Heart Journal. Case Reports*.

[46] Mints YY, Doros G, Berk JL, Connors LH, Ruberg FL (2018). Features of atrial fibrillation in wild-type transthyretin cardiac amyloidosis: a systematic review and clinical experience. *ESC Heart Failure*.

[47] Staerk L, Sherer JA, Ko D, Benjamin EJ, Helm RH (2017). Atrial Fibrillation: Epidemiology, Pathophysiology, and Clinical Outcomes. *Circulation Research*.

[48] Wang TJ, Larson MG, Levy D, Vasan RS, Leip EP, Wolf PA (2003). Temporal relations of atrial fibrillation and congestive heart failure and their joint influence on mortality: the Framingham Heart Study. *Circulation*.

[49] Giancaterino S, Urey MA, Darden D, Hsu JC (2020). Management of Arrhythmias in Cardiac Amyloidosis. *JACC. Clinical Electrophysiology*.

[50] Writing Committee, Kittleson MM, Ruberg FL, Ambardekar AV, Brannagan TH, Cheng RK (2023). 2023 ACC Expert Consensus Decision Pathway on Comprehensive Multidisciplinary Care for the Patient With Cardiac Amyloidosis: A Report of the American College of Cardiology Solution Set Oversight Committee. *Journal of the American College of Cardiology*.

[51] Bukhari S, Khan SZ, Bashir Z (2023). Atrial Fibrillation, Thromboembolic Risk, and Anticoagulation in Cardiac Amyloidosis: A Review. *Journal of Cardiac Failure*.

[52] Lee SR, Choi JM (2024). Is It Time to Expand the Indication of DOAC to Patients With Cardiac Amyloidosis and Atrial Fibrillation?. *International Journal of Heart Failure*.

[53] Nong Q, Liang S, Zhu W, Chen Y, Zhang T (2025). Direct Oral Anticoagulants vs. Vitamin K Antagonists for Atrial Fibrillation in Cardiac Amyloidosis: A Systematic Review and Meta-analysis. *Reviews in Cardiovascular Medicine*.

[54] Cheung CC, Roston TM, Andrade JG, Bennett MT, Davis MK (2020). Arrhythmias in Cardiac Amyloidosis: Challenges in Risk Stratification and Treatment. *The Canadian Journal of Cardiology*.

[55] Gertz MA, Skinner M, Connors LH, Falk RH, Cohen AS, Kyle RA (1985). Selective binding of nifedipine to amyloid fibrils. *The American Journal of Cardiology*.

[56] Rubinow A, Skinner M, Cohen AS (1981). Digoxin sensitivity in amyloid cardiomyopathy. *Circulation*.

[57] Tan NY, Mohsin Y, Hodge DO, Lacy MQ, Packer DL, Dispenzieri A (2016). Catheter Ablation for Atrial Arrhythmias in Patients With Cardiac Amyloidosis. *Journal of Cardiovascular Electrophysiology*.

[58] Barbhaiya CR, Kumar S, Baldinger SH, Michaud GF, Stevenson WG, Falk R (2016). Electrophysiologic assessment of conduction abnormalities and atrial arrhythmias associated with amyloid cardiomyopathy. *Heart Rhythm*.

[59] Donnellan E, Wazni O, Kanj M, Elshazly MB, Hussein A, Baranowski B (2020). Atrial fibrillation ablation in patients with transthyretin cardiac amyloidosis. *Europace: European Pacing, Arrhythmias, and Cardiac Electrophysiology: Journal of the Working Groups on Cardiac Pacing, Arrhythmias, and Cardiac Cellular Electrophysiology of the European Society of Cardiology*.

[60] Dale Z, Chandrashekar P, Al-Rashdan L, Gill S, Elman M, Fischer KL (2022). Routine ambulatory heart rhythm monitoring for detection of atrial arrhythmias in transthyretin cardiac amyloidosis. *International Journal of Cardiology*.

[61] Stables RH, Ormerod OJ (1996). Atrial thrombi occurring during sinus rhythm in cardiac amyloidosis: evidence for atrial electromechanical dissociation.

[62] Donnellan E, Elshazly MB, Vakamudi S, Wazni OM, Cohen JA, Kanj M (2019). No Association Between CHADS-VASc Score and Left Atrial Appendage Thrombus in Patients With Transthyretin Amyloidosis. *JACC. Clinical Electrophysiology*.

[63] El-Am EA, Dispenzieri A, Melduni RM, Ammash NM, White RD, Hodge DO (2019). Direct Current Cardioversion of Atrial Arrhythmias in Adults With Cardiac Amyloidosis. *Journal of the American College of Cardiology*.

[64] Sucker C, Hetzel GR, Grabensee B, Stockschlaeder M, Scharf RE (2006). Amyloidosis and bleeding: pathophysiology, diagnosis, and therapy. *American Journal of Kidney Diseases: the Official Journal of the National Kidney Foundation*.

[65] Wang XF, Li T, Yang M, Huang Y (2024). Periorbital purpura can be the only initial symptom of primary light chain amyloidosis: A case report. *World Journal of Clinical Cases*.

[66] Dejhansathit S, Suvannasankha A (2019). Acquired Factor X Deficiency in Patients With Primary Light Chain Amyloidosis. *Journal of Investigative Medicine High Impact Case Reports*.

[67] Manikkan AT (2012). Factor X deficiency: an uncommon presentation of AL amyloidosis. *Upsala Journal of Medical Sciences*.

[68] Liebman H, Chinowsky M, Valdin J, Kenoyer G, Feinstein D (1983). Increased fibrinolysis and amyloidosis. *Archives of Internal Medicine*.

[69] Lacy SC, Kinno M, Joyce C, Yu MD (2023). Direct Oral Anticoagulants in Patients With Cardiac Amyloidosis: A Systematic Review and Meta-Analysis. *International Journal of Heart Failure*.

[70] Roccatello D, Fenoglio R, Sciascia S, Naretto C, Rossi D, Ferro M (2020). CD38 and Anti-CD38 Monoclonal Antibodies in AL Amyloidosis: Targeting Plasma Cells and beyond. *International Journal of Molecular Sciences*.

[71] Bianchi G, Zhang Y, Comenzo RL (2021). AL Amyloidosis: Current Chemotherapy and Immune Therapy Treatment Strategies: JACC: CardioOncology State-of-the-Art Review. *JACC. CardioOncology*.

[72] Mesquita ET, Jorge AJL, Souza CV, Andrade TRD (2017). Cardiac Amyloidosis and its New Clinical Phenotype: Heart Failure with Preserved Ejection Fraction. *Arquivos Brasileiros De Cardiologia*.

[73] Olivotto I, Udelson JE, Pieroni M, Rapezzi C (2023). Genetic causes of heart failure with preserved ejection fraction: emerging pharmacological treatments. *European Heart Journal*.

[74] Antonopoulos AS, Panagiotopoulos I, Kouroutzoglou A, Koutsis G, Toskas P, Lazaros G (2022). Prevalence and clinical outcomes of transthyretin amyloidosis: a systematic review and meta-analysis. *European Journal of Heart Failure*.

[75] Tanskanen M, Peuralinna T, Polvikoski T, Notkola IL, Sulkava R, Hardy J (2008). Senile systemic amyloidosis affects 25% of the very aged and associates with genetic variation in alpha2-macroglobulin and tau: a population-based autopsy study. *Annals of Medicine*.

[76] Cipriani A, De Michieli L, Porcari A, Licchelli L, Sinigiani G, Tini G (2022). Low QRS Voltages in Cardiac Amyloidosis: Clinical Correlates and Prognostic Value. *JACC. CardioOncology*.

[77] Argirò A, Del Franco A, Mazzoni C, Allinovi M, Tomberli A, Tarquini R (2022). Arrhythmic Burden in Cardiac Amyloidosis: What We Know and What We Do Not. *Biomedicines*.

[78] Mesquita T, Zhang R, Cho JH, Zhang R, Lin YN, Sanchez L (2022). Mechanisms of Sinoatrial Node Dysfunction in Heart Failure With Preserved Ejection Fraction. *Circulation*.

[79] Maloberti A, Ciampi C, Politi F, Fabbri S, Musca F, Giannattasio C (2024). Cardiac amyloidosis red flags: What all the cardiologist have to know. *International Journal of Cardiology. Cardiovascular Risk and Prevention*.

[80] Dorbala S, Vangala D, Bruyere J, Quarta C, Kruger J, Padera R (2014). Coronary microvascular dysfunction is related to abnormalities in myocardial structure and function in cardiac amyloidosis. *JACC. Heart Failure*.

[81] Kolijn D, Kovács Á, Herwig M, Lódi M, Sieme M, Alhaj A (2020). Enhanced Cardiomyocyte Function in Hypertensive Rats With Diastolic Dysfunction and Human Heart Failure Patients After Acute Treatment With Soluble Guanylyl Cyclase (sGC) Activator. *Frontiers in Physiology*.

[82] Franssen C, Chen S, Unger A, Korkmaz HI, De Keulenaer GW, Tschöpe C (2016). Myocardial Microvascular Inflammatory Endothelial Activation in Heart Failure With Preserved Ejection Fraction. *JACC. Heart Failure*.

[83] Saito Y, Nakamura K, Ito H (2021). Molecular Mechanisms of Cardiac Amyloidosis. *International Journal of Molecular Sciences*.

[84] Sinha A, Rahman H, Perera D (2023). Coronary microvascular dysfunction and heart failure with preserved ejection fraction: what are the mechanistic links?. *Current Opinion in Cardiology*.

[85] Netti L, Ioannou A, Martinez-Naharro A, Razvi Y, Porcari A, Venneri L (2024). Microvascular obstruction in cardiac amyloidosis. *European Journal of Heart Failure*.

[86] Aimo A, Bonino L, Castiglione V, Musetti V, Rossetti M, Celi A (2024). Rarefaction of Blood, But Not Lymphatic Capillaries, in Patients With Cardiac Amyloidosis. *JACC. CardioOncology*.

[87] Falk RH, Alexander KM, Liao R, Dorbala S (2016). AL (Light-Chain) Cardiac Amyloidosis: A Review of Diagnosis and Therapy. *Journal of the American College of Cardiology*.

[88] Nguyen HT, Nguyen CTH (2020). Cardiac amyloidosis mimicking acute coronary syndrome: a case report and literature review. *European Heart Journal. Case Reports*.

[89] Nakamura K, Yoshinaga T, Sakyu A, Matsushima A, Yonehara Y, Kojima T (2024). Genetic counselling for at-risk family members with hereditary transthyretin amyloidosis: data from a single-centre study. *Amyloid: the International Journal of Experimental and Clinical Investigation: the Official Journal of the International Society of Amyloidosis*.

[90] Ueda M, Sekijima Y, Koike H, Yamashita T, Yoshinaga T, Ishii T (2020). Monitoring of asymptomatic family members at risk of hereditary transthyretin amyloidosis for early intervention with disease-modifying therapies. *Journal of the Neurological Sciences*.

[91] Schmidt HHJ, Barroso F, González-Duarte A, Conceição I, Obici L, Keohane D (2016). Management of asymptomatic gene carriers of transthyretin familial amyloid polyneuropathy. *Muscle & Nerve*.

[92] Gonzalez-Lopez E, Escobar-Lopez L, Obici L, Saturi G, Bezard M, Saith SE (2022). Prognosis of Transthyretin Cardiac Amyloidosis Without Heart Failure Symptoms. *JACC. CardioOncology*.

[93] Soman P, Garcia-Pavia P, Gillmore JD, Adams D, Conceicao I, Coelho T (2024). Rationale & Design of ACT-EARLY, the Acoramidis Transthyretin Amyloidosis Prevention Trial: Early Detection Using Cardiac Radionuclide Imaging. *Journal of Nuclear Cardiology*.

[94] Ríos-Tamayo R, Krsnik I, Gómez-Bueno M, Garcia-Pavia P, Segovia-Cubero J, Huerta A (2023). AL Amyloidosis and Multiple Myeloma: A Complex Scenario in Which Cardiac Involvement Remains the Key Prognostic Factor. *Life (Basel, Switzerland)*.

[95] Shetty M, Malhotra S (2023). Novel Tracers for the Imaging of Cardiac Amyloidosis. *Journal of Nuclear Medicine Technology*.

[96] Grogan M, Lopez-Jimenez F, Guthrie S, Kumar N, Langevin R, Lousada I (2025). Value of Artificial Intelligence for Enhancing Suspicion of Cardiac Amyloidosis Using Electrocardiography and Echocardiography: A Narrative Review. *Journal of the American Heart Association*.

[97] Bumma N, Kahwash R, Parikh SV, Isfort M, Freimer M, Vallakati A (2022). Multidisciplinary amyloidosis care in the era of personalized medicine. *Frontiers in Neurology*.

[98] Sohn HR, Song BG, Jeong SY, Hong SM, Jung HG, Jung HJ (2011). A Case of Cardiac Amyloidosis Initially Misdiagnosed as Syndrome X. *Cardiology Research*.

[99] George A, McClements B (2015). Cardiac amyloidosis presenting as recurrent acute coronary syndrome with unobstructed coronary arteries: Case report. *Indian Heart Journal*.

[100] Taiwo AA, Alapati L, Movahed A (2019). Cardiac amyloidosis: A case report and review of literature. *World Journal of Clinical Cases*.

[101] De Michieli L, De Gaspari M, Sinigiani G, Lupi A, Vedovelli L, Salvalaggio A (2023). Chest pain in cardiac amyloidosis: occurrence, causes and prognostic significance. *International Journal of Cardiology*.

[102] Mathew V, Olson LJ, Gertz MA, Hayes DL (1997). Symptomatic conduction system disease in cardiac amyloidosis. *The American Journal of Cardiology*.

[103] Longhi S, Quarta CC, Milandri A, Lorenzini M, Gagliardi C, Manuzzi L (2015). Atrial fibrillation in amyloidotic cardiomyopathy: prevalence, incidence, risk factors and prognostic role. *Amyloid: the International Journal of Experimental and Clinical Investigation: the Official Journal of the International Society of Amyloidosis*.

[104] Boufidou A, Mantziari L, Paraskevaidis S, Karvounis H, Nenopoulou E, Manthou ME (2010). An interesting case of cardiac amyloidosis initially diagnosed as hypertrophic cardiomyopathy. *Hellenic Journal of Cardiology: HJC = Hellenike Kardiologike Epitheorese*.

[105] Argirò A, Silverii MV, Burgisser C, Fattirolli F, Baldasseroni S, di Mario C (2024). Serial Changes in Cardiopulmonary Exercise Testing Parameters in Untreated Patients With Transthyretin Cardiac Amyloidosis. *The Canadian Journal of Cardiology*.

[106] Zhang D, Makhni MC, Kang JD, Blazar P (2021). Orthopaedic Manifestations of Amyloidosis. *The Journal of the American Academy of Orthopaedic Surgeons*.

[107] Zhang KW, Vallabhaneni S, Alvarez-Cardona JA, Krone RJ, Mitchell JD, Lenihan DJ (2021). Cardiac Amyloidosis for the Primary Care Provider: A Practical Review to Promote Earlier Recognition of Disease. *The American Journal of Medicine*.

[108] Cheng RK, Maurer MS (2021). Recognition and Implications of Undiagnosed Cardiac Amyloid Patients in HFpEF Trials. *JACC. Heart Failure*.

[109] Fathala A (2020). Incidentally detected cardiac amyloidosis on ^99m^Tc-MDP bone scintigraphy. *Radiology Case Reports*.

[110] Kazi DS, Bellows BK, Baron SJ, Shen C, Cohen DJ, Spertus JA (2020). Cost-Effectiveness of Tafamidis Therapy for Transthyretin Amyloid Cardiomyopathy. *Circulation*.

[111] Lau ATC, DiDomenico RJ, Kim K (2024). Cost-effectiveness of systematic screening and treatment of transthyretin amyloid cardiomyopathy (ATTR-CM) in patients with heart failure with preserved ejection fraction (HFpEF) in United States. *International Journal of Cardiology*.

[112] Ge Y, Pandya A, Cuddy SAM, Singh A, Singh A, Dorbala S (2022). Modeling the Cost and Health Impacts of Diagnostic Strategies in Patients with Suspected Transthyretin Cardiac Amyloidosis. *Journal of the American Heart Association*.

